# Mild Traumatic Brain Injury and Functional Amnesia: When Concussion Becomes a Gateway to Functional Cognitive Disorder

**DOI:** 10.3390/brainsci16030278

**Published:** 2026-02-28

**Authors:** Ioannis Mavroudis, Foivos Petridis, Alin Ciobica, Sotirios Papagiannopoulos, Dimitrios Kazis

**Affiliations:** 1 Institute of Health Sciences, Leeds University, Leeds LS2 9JT, UK; 2 Third Department of Neurology, Aristotle University of Thessaloniki, GR-54124 Thessaloniki, Greece; f_petridis83@yahoo.gr (F.P.); ubic2@otenet.gr (S.P.); dimitrios.kazis@gmail.com (D.K.); 3 Academy of Romanian Scientists, 3 Ilfov, 050044 Bucharest, Romania; 4 Department of Biology, Faculty of Biology, Alexandru Ioan Cuza University of Iasi, Carol I Avenue 20Th A, 700505 Iasi, Romania; 5 “Ioan Haulica” Institute, Apollonia University, Pacurari Street 11, 700511 Iasi, Romania; 6 Center of Biomedical Research, Romanian Academy, Iasi Branch, Teodor Codrescu 2, 700481 Iasi, Romania

**Keywords:** mild traumatic brain injury, functional amnesia, functional cognitive disorder, autobiographical memory, dissociation

## Abstract

Mild traumatic brain injury (mTBI) is typically associated with transient cognitive disturbance, particularly involving attention and new learning, with most patients demonstrating full recovery within weeks. Memory impairment in uncomplicated mTBI generally reflects reversible neurometabolic dysfunction and is limited to a brief period of post-traumatic amnesia and restricted retrograde loss surrounding the injury. However, a subset of patients develop persistent and disproportionate autobiographical memory disturbance that exceeds expected neuroanatomical limits and lacks structural correlates on neuroimaging. In rare but clinically challenging cases, this presentation may resemble extensive retrograde or identity-related amnesia. This review examines functional (dissociative) amnesia emerging after mTBI and proposes that concussion may act as a gateway condition facilitating the development of Functional Cognitive Disorder (FCD) in vulnerable individuals. We differentiate expected post-traumatic memory patterns from atypical selective impairment of autobiographical retrieval and clarify how distinct memory systems—episodic, autobiographical, semantic, and procedural—are differentially affected. We expand the two-hit hypothesis by integrating contemporary neurobiological evidence. The first hit comprises concussion-induced neurometabolic disturbance, glial activation, oxidative imbalance, and transient fronto-limbic dysregulation. The second hit may involve psychological stress, identity threat, maladaptive metacognitive processes, or persistent neuroinflammatory signalling, collectively resulting in functional inhibition of autobiographical memory retrieval despite preserved memory storage. Functional amnesia is conceptualised as a severe phenotype within the spectrum of functional cognitive disorder. We introduce a structured clinician-administered interview (SIFRA) to operationalise diagnostic features and support systematic assessment. This integrative framework reconciles neurological vulnerability with functional network dysregulation and provides a coherent basis for diagnosis and multidisciplinary management of persistent memory disturbance after mTBI.

## 1. Introduction

Mild traumatic brain injury (mTBI), commonly referred to as concussion, is among the most frequent neurological insults worldwide and represents a significant public health burden [1,2,3,4,5]. It accounts for the majority of traumatic brain injuries presenting to emergency departments and commonly results from falls, road traffic accidents, sports injuries, and occupational trauma [1,2]. Although traditionally considered benign and self-limiting, mTBI is increasingly recognised as a heterogeneous condition with variable clinical trajectories [3,4,5].

In most individuals, recovery is rapid and complete. Acute cognitive symptoms—headache, slowed processing, attentional inefficiency, and memory disturbance—typically resolve within days or weeks [3,4]. However, a clinically meaningful minority experience persistent post-concussive symptoms, including subjective cognitive impairment, reduced mental stamina, and ongoing memory complaints that may persist for months or years [4,5]. These symptoms often occur in the absence of abnormalities on conventional structural neuroimaging, generating diagnostic uncertainty for both patients and clinicians.

Memory disturbance has long been central to the clinical characterisation of traumatic brain injury. Classical descriptions emphasised post-traumatic amnesia (PTA) and a limited retrograde amnesia (RTA), usually confined to events immediately preceding injury [6,7,8]. Russell’s seminal work established the relationship between injury severity, PTA duration, and prognosis—a framework that remains foundational in clinical practice [6,7,8]. In mTBI, these disturbances are generally brief and reflect transient network dysfunction rather than permanent neuronal loss [9,10].

Nevertheless, a subset of patients present with memory impairment that exceeds expected neurobiological limits. Some report extensive retrograde amnesia extending months or years before injury, selective loss of autobiographical memory, or even disruption of personal identity [11,12,13,14,15]. Such presentations are difficult to reconcile with lesion-based models, particularly when neurological examination and neuroimaging are unremarkable.

Functional (dissociative) amnesia offers an alternative explanatory framework. It is characterised by impaired access to autobiographical memory in the absence of structural brain damage, neurodegenerative disease, or intoxication [11,12,13,14,15]. Critically, it reflects a disorder of retrieval rather than storage. Patients typically retain semantic knowledge, procedural learning, and the capacity for new memory formation, distinguishing functional amnesia from hippocampal or diencephalic amnesic syndromes [16,17,18].

Over the past two decades, functional amnesia has gained increasing neurobiological credibility. Functional neuroimaging studies demonstrate reproducible alterations in fronto-limbic networks involved in memory retrieval, emotional regulation, and self-referential processing [19,20,21,22]. These findings challenge outdated “organic–psychogenic” dichotomies and instead support a network-based account of memory dysfunction.

Importantly, mTBI itself increases vulnerability to affective disturbance, stress dysregulation, and maladaptive cognitive control. Depression, anxiety, and post-traumatic stress symptoms are common after concussion and are established modulators of memory performance [23,24,25]. This overlap suggests that mTBI may create a neurocognitive milieu in which functional mechanisms are more likely to shape clinical presentation, rather than acting as a sole or sufficient cause of persistent amnesia.

The two-hit hypothesis provides a unifying explanatory model. Originally formulated in the context of functional amnesia, it proposes that a physical insult constitutes a first hit producing neural vulnerability, while a second hit—typically psychological or contextual—precipitates functional disruption of memory retrieval [19,26,27]. Within this framework, mTBI may function as a gateway condition, lowering the threshold for functional cognitive disorders.

Functional cognitive disorder (FCD) has emerged as a clinically meaningful construct describing persistent cognitive complaints driven by dysfunctional cognitive control, attentional misallocation, and maladaptive metacognitive processes rather than structural pathology [28,29,30,31]. Functional amnesia can be conceptualised as a severe and focal manifestation within this broader spectrum, particularly when autobiographical memory systems are preferentially affected.

In this review, we examine the interface between mTBI and functional amnesia, proposing that concussion may act as a gateway to FCD in vulnerable individuals. By integrating evidence from neuropsychology, neurobiology, and clinical neurology, we aim to provide a coherent framework for diagnosis and management of these complex presentations.

Despite increasing recognition of functional amnesia and FCD, structured tools to support diagnosis in routine neurological practice remain limited. Assessment frequently relies on unstructured interviews and exclusionary reasoning, contributing to variability and uncertainty. To address this gap, we introduce the Structured Interview for Functional Retrograde Amnesia (SIFRA), a clinician-administered framework grounded in established theoretical and neurobiological models. SIFRA systematically evaluates autobiographical memory across life periods, preservation of identity, precipitating factors including two-hit patterns, dissociative features, integrity of non-episodic memory systems, and indicators supporting differential diagnosis. It is presented as a practical adjunct to clinical formulation rather than a standalone diagnostic instrument.

## 2. Memory Disturbance After Mild Traumatic Brain Injury: Expected Versus Atypical Patterns

Memory impairment is a defining feature of traumatic brain injury across the severity spectrum and has long been central to injury classification and prognostication [6,7,8,9,10]. In mTBI, disturbance typically manifests as transient impairment of new learning accompanied by brief retrograde amnesia surrounding the injury [1,2,3,4,5]. These changes reflect reversible neurometabolic dysfunction, disrupted network connectivity, and attentional inefficiency rather than permanent structural damage [3,4,5,9,10].

Seminal studies established a relatively orderly relationship between injury severity, duration of post-traumatic amnesia (PTA), and long-term outcome, with mTBI generally associated with rapid recovery [6,7,8]. Neuropsychological assessment in the acute and subacute phases may reveal subtle deficits in attention, processing speed, and working memory; however, these abnormalities usually resolve over time [3,4,5]. Structural neuroimaging in uncomplicated mTBI is typically normal. Although advanced techniques such as diffusion tensor imaging may detect microstructural alterations, correlations with clinical symptoms are inconsistent and do not reliably account for persistent autobiographical memory loss [5,10].

This dissociation becomes particularly apparent in patients reporting extensive retrograde amnesia after mild injury. Atypical presentations include disproportionate retrograde loss extending well beyond the peri-injury period, selective impairment of autobiographical memory, marked context-dependent variability, and preservation of anterograde learning [13,14,15,16]. Some individuals describe loss of emotionally salient life events or disruption of personal narrative despite intact orientation and reasoning [11,12,13,14,15].

Such patterns are inconsistent with established neuroanatomical models of organic amnesia. Severe retrograde amnesia secondary to structural brain injury is ordinarily accompanied by significant anterograde impairment and demonstrable damage to medial temporal or frontal systems [9,10,16]. In their absence, alternative explanatory frameworks must be considered.

Variability is a key distinguishing feature of atypical post-mTBI memory disturbance. Performance often fluctuates according to emotional load, attentional focus, and perceived evaluative threat, improving with distraction and deteriorating under heightened self-monitoring [14,15,28]. This context dependence is difficult to reconcile with fixed structural injury but aligns closely with functional memory disorders.

Psychiatric and psychological factors further shape cognitive outcomes after mTBI. Depression, anxiety, and stress-related symptoms are common and exert well-documented effects on memory encoding and retrieval [23,24,25]. These factors are similarly overrepresented in functional amnesia and functional cognitive disorder (FCD), suggesting shared vulnerability mechanisms rather than coincidental comorbidity [28,29,30,31].

Collectively, these observations indicate that while transient memory impairment is an expected consequence of mTBI, persistent or extensive autobiographical amnesia represents an atypical presentation unlikely to be explained by structural injury alone. Instead, such cases are more coherently understood within a functional framework, in which mTBI acts as a precipitating or permissive factor rather than a sufficient cause of enduring memory dysfunction.

## 3. Functional (Dissociative) Amnesia: Clinical Features and Neurobiological Mechanisms

Functional (dissociative) amnesia is defined by a marked impairment in the retrieval of autobiographical memory that cannot be explained by structural brain damage, neurodegenerative disease, intoxication, or epileptic phenomena [11,12,13,14,15]. In contrast to organic amnesic syndromes—where combined anterograde and retrograde deficits typically arise from medial temporal or diencephalic injury—functional amnesia is characterised by disproportionate impairment of episodic–autobiographical memory with relative preservation of semantic knowledge, procedural learning, and new memory formation [16,17,18]. This selective retrieval disturbance is central to diagnosis.

Clinically, functional amnesia most commonly presents as retrograde loss affecting emotionally salient or identity-defining life periods [11,12,13,14,15]. Patients may report inability to recall personal relationships, occupational history, or major autobiographical events while remaining oriented, articulate, and cognitively intact on formal testing. Anterograde memory is usually preserved or only mildly inefficient, distinguishing functional amnesia from hippocampal amnesia [16,17,18]. In severe cases, autobiographical loss may extend to aspects of personal identity, producing dramatic but neurologically incongruent presentations [11,12,13,14,15].

A defining feature is internal inconsistency. Memory performance often fluctuates across contexts and over time, improving when attention is externally directed and deteriorating under self-monitoring, emotional salience, or perceived evaluative threat [14,15,28]. Such variability is incompatible with fixed structural damage and instead implicates dysfunction in higher-order cognitive control systems governing retrieval.

Neuroimaging studies over the past two decades have provided converging evidence for involvement of fronto-limbic networks mediating autobiographical recall [19,20,21,22]. Altered activation or hypometabolism in right inferolateral prefrontal cortex, anterior cingulate cortex, and orbitofrontal regions—areas exerting top–down regulatory control—has been repeatedly observed [19,20,21]. These findings support models in which memory traces remain intact within hippocampal networks but are rendered functionally inaccessible through excessive prefrontal inhibitory modulation and altered fronto-limbic connectivity [16,17,18,19]. Experimental studies of intentional memory suppression further demonstrate that prefrontal systems can actively inhibit hippocampal activation, particularly for emotionally charged material [22], providing a mechanistic analogue for functional amnesia.

Stress plays a critical modulatory role. Acute and chronic stress influence hippocampal–prefrontal interactions via glucocorticoid-mediated pathways, selectively impairing retrieval while leaving encoding and storage relatively preserved [26,27]. Sustained stress exposure may bias cognitive systems toward avoidance and suppression of emotionally salient autobiographical material, offering a biologically plausible mechanism for selective retrograde loss [16,17,18]. Similar stress-related network dysregulation has been described across functional neurological and cognitive disorders, supporting the conceptualisation of functional amnesia within a broader spectrum of network-level dysfunction [28,29,30,31].

Functional amnesia is frequently misinterpreted as malingering or deliberate fabrication. However, studies examining effort, symptom validity, and performance consistency indicate that most individuals with functional memory disorders do not demonstrate patterns consistent with conscious deception [32,33,34]. Rather, they exhibit genuine distress, functional impairment, and measurable alterations in neural activation patterns. Misclassification as malingering carries significant ethical and clinical consequences, including diagnostic invalidation and therapeutic disengagement [32,33,34].

When functional amnesia occurs following mild traumatic brain injury, these mechanisms acquire particular relevance. mTBI is associated with transient disruption of attentional control, emotional regulation, and network efficiency, even in the absence of structural lesions [1,2,3,4,5,9,10]. Such changes may lower the threshold for retrieval inhibition, particularly in individuals with concurrent psychological stressors, affective vulnerability, or maladaptive illness beliefs [23,24,25]. In this context, concussion does not directly produce functional amnesia but creates a state of neurocognitive vulnerability in which functional retrieval blockade becomes more likely. This formulation reconciles the presence of a genuine neurological insult with the absence of structural correlates and explains why autobiographical memory disturbance may appear severe and disproportionate to injury severity.

## 4. The Two-Hit Hypothesis Applied to Mild Traumatic Brain Injury

The two-hit hypothesis provides a unifying framework for understanding functional amnesia emerging after mild traumatic brain injury [19,26,27]. Rather than viewing concussion and functional symptoms as competing explanations, this model conceptualises mTBI as a permissive condition that interacts with psychological and contextual factors to produce functional disruption of memory retrieval.

The first hit in this model is the mTBI itself. Although concussion does not typically result in macroscopic structural damage, it is associated with transient neurometabolic disturbance, altered network connectivity, and inefficiencies in attentional and executive control systems [1,2,3,4,5,9,10]. These changes are usually reversible but create a period of heightened neural vulnerability during which cognitive control and emotional regulation may be compromised.

Beyond transient neurometabolic disturbance, accumulating evidence indicates that even mild traumatic brain injury initiates a complex glial and inflammatory response [35]. Astrocytes, microglia, and oligodendrocytes form a metabolically integrated syncytium that regulates synaptic homeostasis and energy supply. Following mTBI, microglial activation and astrocytic reactivity may persist beyond the acute phase, contributing to altered hippocampal plasticity and fronto-limbic connectivity. Subtle disruptions in astrocyte-mediated lactate shuttling and oligodendrocyte-supported axonal conduction may prolong network inefficiency even in the absence of structural lesions. In parallel, oxidative stress and oxido-reductive imbalance can impair synaptic transmission and long-term potentiation, mechanisms critical for autobiographical memory retrieval. Emerging evidence also suggests that traumatic stress and head injury may reactivate latent neurotropic viruses or sustain low-grade neuroinflammatory signalling in vulnerable individuals, further modulating memory networks. Thus, the “first hit” may represent not merely transient dysfunction but a period of biologically mediated network vulnerability [36,37,38,39,40,41,42,43].

Concussion also frequently precipitates heightened self-monitoring and concern about cognitive performance. Patients are often encouraged—explicitly or implicitly—to attend closely to memory lapses and cognitive fluctuations, which may be further reinforced by repeated assessment and investigation [3,4,5]. This increased attentional focus can interfere with automatic retrieval processes and amplify subjective cognitive failure.

The second hit is typically psychological or contextual in nature. Contributing factors may include emotional stress, threat to personal or occupational identity, health anxiety, maladaptive illness beliefs, or contextual pressures such as litigation [23,24,25]. Depression, anxiety, and post-traumatic stress symptoms—common after mTBI—further exacerbate prefrontal–limbic dysregulation and impair memory retrieval [23,24,25].

The interaction between these two hits results in functional inhibition of autobiographical memory retrieval despite preserved memory storage (Table 1). Neurobiologically, this state is characterised by excessive top–down inhibitory control from prefrontal regions combined with limbic hyperactivation, biasing memory systems toward suppression and avoidance [19,22,26,27,44]. This mechanism accounts for several otherwise puzzling clinical features, including disproportionate severity, delayed onset, and variability of symptoms.

Certain individuals may be particularly vulnerable to this two-hit interaction. Risk factors include pre-existing anxiety or depressive disorders, prior exposure to psychological trauma, post-traumatic stress symptoms, repeated concussive injuries, high illness anxiety, and situations involving threat to personal or occupational identity. In such contexts, the combination of biological vulnerability and heightened self-monitoring may increase the likelihood that functional mechanisms dominate clinical presentation.

Importantly, the two-hit model explains why the extent of amnesia bears little relationship to biomechanical injury severity or imaging findings [9,10,16]. It also accommodates the observation that memory symptoms may worsen over time as psychological stressors accumulate or maladaptive beliefs become entrenched.

From a theoretical perspective, the two-hit hypothesis aligns with contemporary predictive processing models of brain function, which emphasise the role of expectation, prior beliefs, and top–down modulation in shaping cognitive experience [44,45,46]. Following mTBI, heightened expectation of cognitive impairment may bias memory systems toward perceived failure, reinforcing dysfunctional cognitive patterns and consolidating functional symptoms.

Crucially, this model does not imply that symptoms are “all psychological” or that the injury is irrelevant. Rather, it integrates the genuine neurological impact of concussion with the powerful modulatory effects of stress, attention, and belief systems. In doing so, it provides a non-dualistic, neurobiologically plausible explanation for functional amnesia after mTBI.

## 5. Functional Amnesia as a Subtype of Functional Cognitive Disorder

Functional cognitive disorder (FCD) has emerged as a clinically meaningful framework for persistent cognitive complaints that are incongruent with neurodegenerative disease, focal brain injury, or progressive neurological pathology [28,29,30,31]. Patients typically report prominent subjective difficulties—most often affecting memory, attention, and executive function—that are disproportionate to objective neuropsychological findings. These symptoms are understood to arise from dysfunctional cognitive control, maladaptive attentional allocation, and disturbed metacognitive processes rather than degradation of stored information.

Within this framework, functional amnesia may be conceptualised as a focal and severe phenotype of FCD. Whereas many individuals with FCD describe diffuse inefficiency, “brain fog,” or forgetfulness, functional amnesia represents a circumscribed impairment of autobiographical memory retrieval [11,12,13,14,15,16,17,18]. Both conditions share core phenomenological features, including internal inconsistency, context dependence, heightened self-monitoring, and symptom amplification under emotional or evaluative pressure [28,29,30,31].

Autobiographical memory occupies a distinctive role in cognitive architecture, integrating episodic recall with emotional salience, self-referential processing, and identity continuity [47]. In FCD, excessive monitoring of perceived cognitive failure disrupts normally automatic processes, leading to subjective inefficiency and reduced confidence [28,29,30,31]. When this dysregulation selectively affects autobiographical retrieval—particularly under conditions of identity threat, stress, or trauma—the presentation may evolve into functional amnesia [11,12,13,14,15,16,17,18].

The relationship between FCD and functional amnesia becomes especially relevant following mild traumatic brain injury. Persistent post-concussive cognitive symptoms are increasingly recognised as functional when they lack structural correlates and demonstrate variability and inconsistency [3,4,5,28,29,30,31]. Within this continuum, functional amnesia can be understood as lying at the severe end of post-concussive cognitive disturbance, extending from expected recovery through post-concussion symptoms and FCD to profound autobiographical memory disruption (Table 2). This dimensional perspective avoids dichotomous classification and situates functional amnesia within a broader spectrum of network-level cognitive dysregulation.

Conceptualising functional amnesia as a subtype of FCD carries important diagnostic implications. It encourages clinicians to move away from rigid organic–psychogenic dichotomies and toward a formulation-based approach grounded in symptom mechanisms [28,29,30,31]. This perspective also reduces the risk of misdiagnosis as early neurodegenerative disease, malingering, or irreversible brain injury—misattributions that can significantly worsen prognosis.

From a prognostic perspective, functional amnesia generally carries a more favourable outlook than structurally mediated amnesia, provided appropriate formulation and intervention occur early [13,14,15,16,17,18,28,29,30,31]. Prolonged litigation, adversarial assessment, and repeated emphasis on brain damage may entrench symptoms and reduce the likelihood of recovery.

Key distinguishing features between organic and functional amnesia following traumatic brain injury are summarised in Table 3.

## 6. A Structured Clinical Interview for the Assessment of Functional Retrograde Amnesia

Despite increasing recognition of functional (dissociative) amnesia, there remains a lack of structured, clinically practical tools to support diagnosis in routine neurological settings. Diagnosis is often based on expert clinical judgement, exclusion of alternative causes, and qualitative assessment of autobiographical memory, which may contribute to diagnostic uncertainty and inter-clinician variability [11,12,13,14,15,16,17,18,19,28,29,30,31].

To address this gap, we have developed a Structured Interview for Functional Retrograde Amnesia (SIFRA), a clinician-administered assessment designed to operationalise key diagnostic features of functional amnesia described in the literature, particularly those articulated by Markowitsch and Staniloiu [16,17,18,19]. The key diagnostic domains assessed in the Structured Interview for Functional Retrograde Amnesia (SIFRA) are summarised in Table 4.

### 6.1. Conceptual Framework

The interview is explicitly grounded in established theoretical and neurobiological models of functional amnesia. Its structure reflects five core principles repeatedly identified in prior work:

Disproportionate retrograde autobiographical memory impairment with relative preservation of other memory systems

Absence or reversal of Ribot’s temporal gradient, distinguishing functional from organic amnesia

Preservation of personal semantic and procedural knowledge despite episodic-autobiographical impairment

Association with psychological stressors or a two-hit pattern, rather than congruent structural brain injury

Presence of dissociative features and variability across contexts

These principles align with contemporary formulations of functional cognitive disorder and predictive processing models of functional neurological symptoms [28,29,30,31,35,36,37].

### 6.2. Structure of the Interview

The SIFRA is organised into six modular domains, each targeting a distinct diagnostic dimension of functional retrograde amnesia:

#### 6.2.1. Autobiographical Memory Interview (AMI Module)

This module adapts established autobiographical memory assessment approaches to systematically examine memory across life periods (childhood, adolescence, early adulthood, mid-adulthood). For each epoch, personal semantic memory and episodic-autobiographical recall are scored separately, allowing detection of selective autobiographical impairment and loss of temporal gradient.

This structure directly operationalises the classical distinction between organic and functional amnesia described by Ribot and subsequent authors [6,7,8,16,17,18].

#### 6.2.2. Personal Identity Preservation

This domain assesses factual self-knowledge, including name, date of birth, family members, occupation, and current address. Loss of such information is uncommon in organic retrograde amnesia but frequently reported in dissociative presentations [11,12,13,14,15,16,17,18]. Preservation of identity strongly argues against functional amnesia and supports alternative diagnoses.

#### 6.2.3. Precipitating Factors and the Two-Hit Pattern

This module systematically records physical precipitants (e.g., head trauma, loss of consciousness) and psychological precipitants (recent stressors, childhood trauma, psychiatric history). The presence of a two-hit pattern—early vulnerability followed by a later trigger—is explicitly documented, reflecting the model proposed by Markowitsch and colleagues and extended in the present review [19,26,27].

#### 6.2.4. Dissociative Features Checklist

This checklist captures commonly associated dissociative phenomena, including depersonalisation, derealisation, emotional blunting, fugue-like behaviour, and overgeneral memory. These features are not required for diagnosis but increase diagnostic confidence when present [11,12,13,14,15,28,29,30,31].

#### 6.2.5. Memory Systems Preservation (Tulving-Based Module)

Drawing on Tulving’s model of multiple memory systems, this section evaluates procedural, priming, perceptual, semantic, and episodic-autobiographical memory. Functional amnesia is characterised by selective impairment of episodic-autobiographical memory with preservation of other systems, a pattern rarely observed in structural amnesia [9,16,17,18,47,48].

#### 6.2.6. Differential Diagnosis Indicators

The final module explicitly contrasts organic indicators (structural lesions, congruent neurological deficits, preserved Ribot gradient) with dissociative indicators (lack of gradient, identity disturbance, sudden onset after stress, variability). This structured comparison is intended to support transparent clinical reasoning rather than replace it.

#### 6.2.7. Clinical Interpretation and Use

The SIFRA is not intended as a psychometric screening test or standalone diagnostic instrument. Rather, it is designed to function as a structured clinical aid that enhances consistency, transparency, and documentation in the assessment of suspected functional retrograde amnesia.

Findings should be interpreted in conjunction with neurological examination, neuroimaging, neuropsychological testing, and longitudinal clinical observation. The tool is particularly useful in:

Neurology and neuropsychiatry clinics;

Post-concussion and FND services;

Complex diagnostic cases;

Medicolegal assessments requiring explicit reasoning.

#### 6.2.8. Future Validation

Structured evaluation of autobiographical memory has important therapeutic implications. Clarifying the preservation of anterograde learning and non-episodic memory systems can reduce patient anxiety regarding neurodegeneration and irreversible brain damage. Explicit demonstration of variability and context dependence may support psychoeducation regarding retrieval mechanisms and facilitate engagement with rehabilitation strategies. Early, transparent formulation reduces diagnostic uncertainty, prevents unnecessary investigations, and may improve prognosis by addressing maladaptive illness beliefs at an early stage.

At present, the SIFRA represents a theoretically grounded, expert-informed assessment framework. Future work will focus on formal validation, including inter-rater reliability, construct validity, and comparison with established autobiographical memory measures. Prospective studies examining its utility in post-concussive populations are warranted.

## 7. Management and Rehabilitation Principles

The management of functional amnesia following mTBI requires a multidisciplinary, formulation-driven approach that addresses cognitive, emotional, and behavioural factors simultaneously. Traditional memory rehabilitation strategies designed for structural amnesia are often ineffective when applied in isolation to functional disorders.

Psychoeducation is the cornerstone of treatment. Patients benefit from clear explanations that their memories are not lost but temporarily inaccessible due to disrupted control mechanisms [28,29,30,31]. Demonstrating preserved learning capacity and variability of performance can help restore cognitive confidence and reduce anxiety-driven retrieval inhibition.

Formulations that integrate the neurological impact of concussion with the role of stress, attention, and expectation are more likely to be accepted and facilitate engagement with rehabilitation. Avoiding language that implies blame or fabrication is essential.

Cognitive-behavioural approaches targeting health anxiety, catastrophic interpretation of cognitive lapses, and maladaptive avoidance behaviours are effective in functional cognitive disorders [26,27]. Techniques aimed at reducing attentional capture and encouraging external focus may indirectly improve memory access.

Where emotional trauma or identity disruption is prominent, trauma-informed psychological interventions may be indicated [23,24,25].

Rehabilitation should prioritise functional goals rather than performance on formal memory tests. Gradual re-engagement with work, social roles, and meaningful activities supports recovery by shifting attention away from internal monitoring and toward external engagement [28,29,30,31]. Family education is also crucial, as over-accommodation may inadvertently perpetuate symptoms.

## 8. Conclusions and Future Directions

Functional amnesia following mild traumatic brain injury represents a disorder of memory access arising from disrupted interaction between neural vulnerability and psychological stress. Persistent autobiographical memory disturbance after mild traumatic brain injury should not be presumed to reflect structural damage in the absence of neuroanatomical correlates but instead warrants careful evaluation for functional retrieval dysfunction. Conceptualising functional amnesia as a subtype of functional cognitive disorder provides a coherent framework that integrates neurobiology, psychology, and clinical observation.

Within this framework, concussion is best understood not as the sole cause of persistent amnesia, but as a gateway condition that lowers the threshold for functional cognitive disruption in vulnerable individuals. Early recognition, clear formulation, and targeted intervention offer the best opportunity for recovery and prevention of long-term disability.

Future research should focus on longitudinal outcomes, biomarkers of recovery, and development of evidence-based treatment pathways tailored to post-concussive functional cognitive syndromes.

## Figures and Tables

**Table 1:** Two-Hit Model of Functional Amnesia After Mild Traumatic Brain Injury.

Component	Description
**First hit**	Mild traumatic brain injury causing neurometabolic disturbance, glial activation, oxidative imbalance, and transient fronto-limbic network vulnerability
**Second hit**	Psychological or contextual stressors (acute stress, identity threat, health anxiety, illness beliefs, litigation)
**Mechanism**	Excessive top–down inhibitory control and fronto-limbic dysregulation leading to impaired memory retrieval
**Memory status**	Memory traces intact; access to autobiographical memory functionally blocked
**Clinical outcome**	Functional retrograde amnesia or functional cognitive disorder

**Table 2:** Continuum of Cognitive Outcomes After Mild Traumatic Brain Injury.

Stage	Clinical Characteristics	Structural Brain Damage
Normal recovery	Transient cognitive symptoms resolving within weeks	Absent
Post-concussion symptoms	Persistent subjective cognitive complaints	Absent or minimal
Functional cognitive disorder	Disproportionate cognitive complaints with variability and inconsistency	Absent
Functional retrograde amnesia	Severe autobiographical memory impairment with preserved learning	Absent

**Table 3:** Distinguishing Organic and Functional Retrograde Amnesia.

Feature	Organic Retrograde Amnesia	Functional (Dissociative) Retrograde Amnesia
Structural lesion	Present	Absent
Ribot’s temporal gradient	Preserved	Absent or reversed
Anterograde memory	Impaired	Preserved
Autobiographical memory	Proportionate loss	Disproportionate loss
Personal identity	Preserved	Frequently impaired
Variability	Low	High
Prognosis	Limited recovery	Potentially reversible
Neuroimaging	Structural lesion present	Normal structural imaging

**Table 4:** Domains Assessed in the Structured Interview for Functional Retrograde Amnesia (SIFRA).

Domain	Purpose
Autobiographical memory interview	Assess episodic and personal semantic memory across life periods
Personal identity preservation	Evaluate factual self-knowledge (name, family, occupation, address)
Precipitating factors	Identify physical and psychological triggers, including two-hit patternsd
Dissociative features	Document depersonalisation, derealisation, emotional blunting, fugue-like features
Memory systems preservation	Assess procedural, priming, semantic, and episodic-autobiographical memory
Differential diagnosis indicators	Contrast features supporting organic versus functional aetiology

Note: SIFRA is intended to support clinical formulation and documentation, not to function as a standalone diagnostic instrument.

## Data Availability

No new data were created or analysed in this study.
